# Development of Novel Gluten-Free Sausage Based on Chickpea, Corn Flour, and HPMC

**DOI:** 10.1155/2022/3616887

**Published:** 2022-05-31

**Authors:** Sedigheh Yazdanpanah, Samaneh Ansarifard, Mahsa Hasani

**Affiliations:** Department of Food Science and Technology, Kazerun Branch, Islamic Azad University, Kazerun, Iran

## Abstract

The present work was carried out to study the effectiveness of substitution of wheat flour with different levels of chickpea flour, corn flour, and hydroxypropyl methylcellulose (HPMC) for the production of gluten-free sausages. The prepared sausages were subjected to physicochemical, color, texture, thermal, and sensory analyses 24 h after production. Proximate chemical composition indicated that the protein content was raised by the addition of chickpea flour. The result of thermal analysis indicated that the addition of chickpea flour increased To, Tp, Tf, and Δ*H* and decreased ∆*W*_1_. Textural properties revealed that hardness was higher in samples containing chickpea flour and adhesiveness, chewiness, and gumminess were lower in samples with chickpea flour and corn flour. Sensorial properties showed that there were no significant differences between samples except the sample containing 6% chickpea flour. Based on the obtained results, it seems that the sample containing 4% corn flour, 6% chickpea flour, and 0.3% HPMC had the best formulation.

## 1. Introduction

Today, consumers demand food products, which can improve their health status and life quality as well as prevent diseases [1]. This increasing demand was the driving force for the food industry to produce foods with certain functional properties to satisfy the consumer's needs [2].

Different meat products contain 15-35% fat, and so, they are categorized as high fat foods. Due to the high consumption of these products, they influence the population diet considerably. In recent years, efforts have been made to reduce the fat content of emulsified meat products in order to improve the healthy state of consumers [1]. One susceptible group of consumers that need special food products is people with celiac disease. To prepare gluten-free meat products for this group of people, gluten must be completely removed from their diet and replaced with refined wheat flour and other seed flours [3, 4]. Few works have been done to produce gluten-free meat products. de Carvalho et al. [5] produced gluten-free low-fat chicken nuggets by using amaranth flakes in the formulation. Devatkal et al. [6] produced gluten-free low-fat chicken nuggets by using sorghum flour in the formulation. Romero et al. [7] prepared gluten-free fish (*Pseudoplatystoma corruscans*) patties with rice, corn, amaranth, or quinoa. Kerimoğlu and Serdaroğlu [8] produced gluten-free meat products by rice flour, sorghum flour, pearl millet flour, amaranth, or quinoa flour.

Different attributes of a food product including function, composition, and rheology affect the food quality. Improving food quality and formulation needs information regarding the properties of its ingredients. Rheological and textural properties of a meat product are among the main factors influencing consumer's acceptance. Hydrocolloids (starch, carrageenan, gellan, locust bean gum, etc.) are among the main ingredients in meet products that develop rheological and textural properties [9].

Chickpea flour is containing proteins such as albumins and globulins. The technofractional properties of proteins confirm that the products of chickpea processing can be used in the technology of cooking foods with a low content of gluten. Also, it contains phosphorus 290 mg/100 g, potassium, and magnesium 126 mg/100 g. For the chickpea varieties studied, high content of essential fatty acids, such as linolenic 30%, linoleic 60%, and oleic 23-28%, is a characteristic [10]. Corn flour is a type of flour milled from dried whole corn kernels. It is considered a whole grain flour because it contains the corn's hull, germ, and endosperm. Corn flour is usually yellow, but it can also be white or blue, depending on the variety of corn used. Corn flour is a thickening agent most often used to make marinades, sauces, dressings, soups, gravies, and some desserts. It can be used for gluten-free products. Hydroxypropyl methylcellulose (HPMC) is a nonionic cellulose ether after a series of etherification using a natural polymer material cellulose as raw material. It is an odorless, nontoxic white powder that swells in cold water into a clear or slightly turbid colloidal solution. It has the characteristics of thickening, dispersing, emulsifying, film-forming, suspension, adsorption, surface activity, moisture retention, and protective colloid [11].

There are limited works on using chickpea flour and corn flour in the formulation of meat products. The aim of the present work was to evaluate the effects of the addition of chickpea flour and corn flour into gluten-free sausage batter and then investigate the physicochemical, textural, and sensorial attributes of the final sausage products.

## 2. Material and Methods

Chickpea flour, corn flour, and wheat flour were purchased from Kesht-e-Sabz Co. (Shiraz, Iran). Hydroxypropyl methylcellulose (HPMC) was prepared by Adonis Gol Darou Co. (Shiraz, Iran). Fresh cow meat was purchased from a local butcher shop in Kazeroun (Iran). All other used chemical materials were of analytical grade.

### 2.1. Proximate Properties of Raw Materials

The chemical composition of different flour types (corn flour, chickpea flour, and wheat flour) including protein, ash, and moisture contents was determined based on the ISO 1442:1997, ISO 936:1998, and ISO 937 standard methods, respectively. A.O.C.S. Official Procedure Am 5-04 (A.O.C.S., 2005) was used as the guideline to measure fat content. All the measurements were carried out in triplicate [12].

### 2.2. Sample Preparation

The connective tissues and excessive fat were removed from the cow meat before use, and then, a meat grinder (5 mm plate, PU 200, Germany) was used to grind the meat. The ground meat (1.6 kg) and the other ingredients in the formulation including ice (0.4 kg), back fat (0.4 kg), L-ascorbic acid (0.02%), sodium chloride (1.5%), and sodium nitrite (0.01%) were mixed in a cutter (K326AC8, Germany) for 30 min. Different amounts of wheat flour (in the control sample), HPMC, corn flour, and chickpea flour were also added to the five formulations (Table 1).

Three batches of each formulation were produced at different times on the same day. Before producing the sausage samples, the batters were kept in a refrigerator (4°C) for half a day and then stuffed in waterproof plastic casings (0.4 kg batter, 60 mm diameter) using a stuffing machine (VF628 Germany). The samples were heated in a water bath at 85°C for 45 min, and after that, the sausages were cooled with tap water for 30 min. The samples were dried and then kept in a refrigerator before use (24 h) [13]. For each type of formulation, ten sausages were produced.

#### 2.2.1. Proximate Composition of Samples

The chemical composition (protein, ash, moisture, and fat contents) of the produced sausages was determined based on the methods mentioned in Section 2.1. All measurements were done in triplicate [12].

#### 2.2.2. pH

To measure the pH value, each type of sausage sample was mixed with deionized water and then homogenized using a mixer. A pH meter (PL500, Taiwan) with a glass electrode (HI 1131P) and temperature electrode (HI 7669/2 W) was used to determine the pH of the obtained suspension of sausage at 25°C [12].

#### 2.2.3. Color Properties

The color properties of the sausage samples were determined based on the CIE system. *L*∗, *a*∗, and *b*∗ in this system show lightness (+: redness and -: greenness and +: yellowness and -: blueness), respectively. Four random points from the surface of the sausage slices were chosen for color measurements. A Minolta CR-400 colorimeter (Konica Minolta Sensing Inc., Japan) with a 10° observation angle, illuminant D65, and spectral reflectance included as calibration modes were used for this purpose [14].

#### 2.2.4. DSC Analysis

The thermal properties of the prepared sausages were evaluated based on the method of Tongnuanchan et al. [15]. Briefly, 5 mg of each sample was accurately weighed in a pan at 50% specific relative humidity (HR) and then sealed to prevent moisture penetration and placed in a differential scanning calorimeter (Perkin-Elmer, Beaconsfield, UK) and heated. The temperature was increased from 30°C to 250°C (10°C/min heating rate) under a nitrogen atmosphere at a flow rate of 20 mL/min. After the first scan, a second scan was carried out in the same way, followed by quench-cooling of the sample.

#### 2.2.5. TGA

Thermogravimetric analysis was conducted based on the method of Tongnuanchan et al. [15]. The sausage samples were scanned during heating from 25°C to 300°C (10°C/min) under nitrogen atmosphere (50 mL/min flow rate).

#### 2.2.6. Texture Analysis

Texture Analyzer CT3 (Brookfield AMETEK, Middleboro, Massachusetts, USA) and TPA test were used to investigate different textural properties of sausage samples including hardness, springiness, adhesiveness, chewiness, and gumminess. All measurements were performed on the same day in triplicate [16]. The sample was compressed to 15 mm penetration depth with 2 mm/s pretest speed and 2 mm/s test speed using a cylinder probe of 25 mm diameter.

#### 2.2.7. Sensorial Properties

To evaluate the sensorial properties of sausage samples including texture, taste, color, appearance, and total acceptance, a seven-point hedonic test (from score 1: dislike very much to score 7: like very much) was used. Ten trained panelists (5 men and 5 women) were chosen from the staff and students in Islamic Azad University of Kazeroun (Department of Food Science and Technology) in the age range of 18-32 years. Samples (two slices of each sausage sample with 0.3 cm thickness and 4 cm diameter) with a three-digital code were given to the panelists. The panelists evaluated samples in standard booths with fluorescence light. Salty crackers and water at ambient temperature were also provided for palate cleaning [14].

### 2.3. Statistical Analysis

All the mentioned experiments were carried out in triplicate. Results were reported as the mean ± standard deviation. To determine significant differences (*P* < 0.05) between different sausage formulations, one-way ANOVA was performed. The Duncan test was used to compare the means.

## 3. Results and Discussion

### 3.1. Chemical Properties of Raw Materials

The results of the chemical composition (protein, ash, fat, and moisture content) of chickpea flour, corn flour, and wheat flour are presented in Table 2. The results showed high levels of fat content (5.40%) and moisture content (7.72%) in corn flour and high contents of protein (17.91%) and ash (1.90%) in chickpea flour. Our results were similar to results reported in other researches in terms of chemical composition. Kaur Sidhu et al. [17] reported that chickpea flour had 21.8% protein and 6% total fat. Díaz et al. [18] reported that based on the proximate chemical analyses, chickpea flour had 12.92% moisture content, 6.14% lipid, 19.41% protein, and 3.21% ash. Dongmo et al. [19] reported that proximate chemical analyses of corn flour samples showed 7.08% moisture content, 2.39% lipid, 11.37% protein, and 1.95% ash. Xue et al. [20] reported that corn flour had 10.92% protein, 11.95% moisture content, 0.41% ash, and 1.92% total fat.

### 3.2. Chemical Properties of Samples

The results of chemical composition (protein, ash, fat, and moisture content) and pH of the gluten-free sausages with different formulations are presented in Table 3. The obtained results showed that the protein level increased (*P* < 0.05) by adding chickpea flour. This result is in line with the high protein content (17.91%) of chickpea flour compared to the other flours. Sanwo et al. [21] reported that substituting wheat flour with rice flour in beef sausage increased the protein content. Investigating chemical composition revealed that the moisture content and ash content of gluten-free samples were higher than the control sample. This result is confirmed by the high moisture content and ash content of chickpea flour and corn flour compared to wheat flour. A similar result was reported by Pires et al. [22] after the addition of chia flour to bologna-type sausages. They reported that the addition of chia flour increased ash content. dos Santos Alves et al. [14] indicated that adding green banana flour to bologna-type sausages increased moisture content of the samples. The results also showed that there were no significant differences between fat content and pH value of different samples. It is due to the type of ingredient and level of addition. The pH of the samples ranged from 6.06 to 6.13 which are acceptable values for sausage samples [23]. dos Santos Alves et al. [14] showed that the addition of green banana flour to the formulation of bologna-type sausages had no any significant effect on the pH value of the samples.

### 3.3. Color Properties

The color property is one of the main aspects of meat products. In addition to the pigments, the structure and composition of meat products have important roles in their color. The color of fresh meat is an indicator of its quality and can promote consumers to buy it. The results of color properties of the different gluten-free sausages are shown in Table 4. It was revealed that the *L*∗ value decreased (*P* < 0.05) by adding chickpea flour. This result is in accordance with the lower *L*∗ value in chickpea flour compared to the other flour types. Pereira et al. [24] reported that the addition of rice flour significantly decreased *L*∗ value of sausage. In a research, it was reported that by adding 10% rice flour, the *L*∗ value of duck and pork sausages decreased considerably [25]. Decreasing the *L*∗ value after the addition of 5% glutinous rice flour to meat patties was also reported by Gao et al. [26]. These results are in line with our result that demonstrated a lower *L*∗ value in sausages containing chickpea flour. The results also showed that there were no significant differences between *a*∗ value and *b*∗ value of different samples. Pereira et al. [24] reported that the addition of rice flour had no significant effect on the *a*∗ value of sausage. Sirini et al. [27] reported that the addition of chestnut flour did not affect *a*∗ and *b*∗ values of dry-cured meat sausages significantly.

### 3.4. DSC Analysis

The results of thermal properties of the different gluten-free sausages, which can be observed in Table 5, showed that To, Tp, Tf, and Δ*H* increased (*P* < 0.05) by adding chickpea flour. The significant differences between the control and other samples were because of the presence of gluten in the control sample. During thermal processing of starch, its crystalline structure is melted, and the small molecules such as amylose are removed from the granules. In wheat flour, gluten surrounds the starch granules [28]. The insoluble gluten and its interaction with water create a strong matrix. The granules of corn and chickpea are gluten-free and show different behavior in comparison to wheat granules. In corn flour and chickpea flour, amylose chains were exited from granules during the cooking process, and so, the final products had weak gel structures. Based on researches, hydrocolloids can interact with starch molecules and improve the texture of meat products [28]. Based on our results, HPMC was a good substitute for gluten. Adding hydrocolloid in the formulation of samples containing starch granules created a layer of hydrocolloid around the granules and decreased leaching of amylose chains. Sivaramakrishnan et al. [29] showed that the addition of HPMC into rice flour improved the rheological properties of bread samples. The effect of protein content on the thermal properties of starch was reported by Mohamed and Rayas-Duarte [30]. They showed that protein could increase the onset temperature of starch samples. The results of TGA parameters of different gluten-free sausages are shown in Table 6. It was observed that ∆*W*_1_ decreased (*P* < 0.05) by adding chickpea flour and ∆*W*_2_ was higher (*P* < 0.05) in the samples containing chickpea flour and corn flour compared to the control sample. The addition of chickpea flour also increased ∆*W*_3_ (*P* < 0.05). The lowest amount of free water (∆*W*_1_) and the highest amount of bonding water (∆*W*_2_ and ∆*W*_3_) in the sample with 8% chickpea flour were related to the higher protein content and the presence of HPMC. Similar results were also reported by Verbeken et al. [31]. The effects of protein content on the free water and bonding water were also reported by Mohamed and Rayas-Duarte [30].

### 3.5. Textural Properties

Rheological evaluations can be divided into the sensory method and the instrumental method. In the present study, we used the instrumental method (TPA) in order to investigate the textural properties of the samples. The results of textural properties of different gluten-free sausages are presented in Table 7. Hardness represents the maximum force of the first compression cycle. The results revealed that the hardness value increased (*P* < 0.05) by adding chickpea flour. Pereira et al. [24] reported that the addition of rice flour increased the hardness of emulsified sausage. Interactions between meat and nonmeat ingredients in emulsified meat products can result in considerable changes in the texture and so the final properties of the gel [32]. Similar to our results, other researches demonstrated that the addition of plant protein in the formulation of sausage increased the hardness value of the sample [33, 34]. However, lower hardness in the meat batter after increasing protein concentration to 10% was reported by Ali et al. [25]. Adhesiveness is the required force to separate the probe from the sample by the first push. The evaluation of textural properties indicated that the adhesiveness, chewiness, and gumminess values were higher in the samples containing chickpea flour and corn flour compared to the control sample. Comparing the sausages containing chickpea flour with those containing corn flour showed significantly lower adhesiveness, chewiness, and gumminess values. The observed differences could be related to different protein contents in chickpea flour and corn flour. Cerón-Guevara et al. [35] reported that the addition of *Agaricus bisporus* flour and *Pleurotus ostreatus* flour improved the chewiness and gumminess of Frankfurter sausages. Choe et al. [13] reported that increasing winter mushroom powder content in the formulation of emulsion-type sausages reduced chewiness and gumminess. Springiness represents the returning speed of a sample to its initial or unchanged condition after disposal of the deformative force. The obtained results showed that the addition of chickpea flour had no significant effect on springiness.

### 3.6. Sensorial Properties

Evaluation of the different sensory attributes of the produced sausages including color, taste, texture, appearance, and consumer acceptance was carried out by ten panelists, and the results are shown in Table 8. It was revealed that there were no significant differences between the samples except that one containing 8% chickpea flour. This sample obtained the lowest scores for color, taste, texture, and overall acceptance. The overall acceptance scores of all samples were between 4.4 and 5.6. Sirini et al. [27] evaluated the impact of chestnut flour on different sensorial properties of dry-cured meat sausages. They reported that the addition of chestnut flour had no significant effects on the color and taste of the samples. Sanwo et al. [21] investigated the effect of substitution of wheat flour with rice flour at different levels on the sensorial properties of beef sausage. They observed significant differences only at high levels of substitution. Leonard et al. [36] reported that incorporating roasted lupin flour into the formulation of beef sausages had no significant effects on the appearance and aroma of the products.

## 4. Conclusion

Substitution of wheat flour with chickpea flour and corn flour caused higher protein levels and lower lightness in the resultant gluten-free sausages. Increasing the chickpea flour level in the formulation improved the thermal stability and textural properties of the samples. Free water content significantly decreased and hardness increased by increasing chickpea flour content. The results of the sensory evaluation showed that chickpea flour could be used in the formulation of gluten-free sausages up to 6% of the total flour composition with no considerable changes in consumer acceptability. Substituting wheat flour with chickpea flour at levels higher than 8% had a negative effect on the acceptability of the products in terms of color, taste, texture, and overall parameters.

## Figures and Tables

**Table 1:** Formulations of flour.

		Wheat flour (%)	Corn flour (%)	Chickpea flour (%)	HPMC (%)
1	Control (wheat flour)	10.3	—	—	—
2	C (8%)-Ch (2%)+HPMC (0.3%)	—	8	2	0.3
3	C (6%)-Ch (4%)+HPMC (0.3%)	—	6	4	0.3
4	C (4%)-Ch (6%)+HPMC (0.3%)	—	4	6	0.3
5	C (2%)-Ch (8%)+HPMC (0.3%)	—	2	8	0.3

**Table 2:** Chemical analysis of chickpea flour, corn flour, and wheat flour.

Sample	Ash (%)	Moisture content (%)	Fat (%)	Protein (%)
Chickpea flour	1.90 ± 0.12^A^	6.50 ± 0.11^B^	3.70 ± 0.13^B^	17.91 ± 1.23^A^
Corn flour	0.68 ± 0.34^B^	7.72 ± 0.26^A^	5.40 ± 0.21^A^	10.22 ± 0.67^B^
Wheat flour	1.00 ± 0.19^B^	6.15 ± 0.07^C^	1.80 ± 0.08^C^	8.10 ± 0.23^C^

^∗^Data represent the mean ± standard deviation of three independent replications. ^∗∗^Different capital letters in each column indicate significant differences (*P* < 0.05).

**Table 3:** Chemical properties of control sausage and gluten-free sausages modified by chickpea flour, corn flour, and HPMC.

Sample	Protein (%)	Fat (%)	Moisture content (%)	Ash (%)	pH (%)
Control (wheat flour)	14.40 ± 0.11^E^	14.12 ± 0.22^A^	57.06 ± 0.11^B^	2.10 ± 0.12^B^	6.13 ± 0.11^A^
C (8%)-Ch (2%)+HPMC (0.3%)	15.01 ± 0.00^D^	14.54 ± 0.13^A^	58.05 ± 0.22^A^	2.24 ± 0.11^AB^	6.11 ± 0.07^A^
C (6%)-Ch (4%)+HPMC (0.3%)	15.22 ± 0.01^C^	14.43 ± 0.15^A^	58.17 ± 0.34^A^	2.35 ± 0.13^A^	6.08 ± 0.13^A^
C (4%)-Ch (6%)+HPMC (0.3%)	15.45 ± 0.05^B^	14.30 ± 0.19^A^	58.25 ± 0.08^A^	2.43 ± 0.11^A^	6.07 ± 0.08^A^
C (2%)-Ch (8%)+HPMC (0.3%)	15.60 ± 0.03^A^	14.22 ± 0.20^A^	58.52 ± 0.12^A^	2.48 ± 0.15^A^	6.06 ± 0.05^A^

C (8%)-Ch (2%): 8% corn flour and 2% chickpea flour; C (6%)-Ch (4%): 6% corn flour and 4% chickpea flour; C (4%)-Ch (6%): 4% corn flour and 6% chickpea flour; C (2%)-Ch (8%): 2% corn flour and 8% chickpea flour. ^∗^Data represent the mean ± standard deviation of three independent replications. ^∗∗^Different capital letters in each column indicate significant differences (*P* < 0.05).

**Table 4:** Color properties of control sausage and gluten-free sausages modified by chickpea flour, corn flour, and HPMC.

Sample	*L*∗	*a*∗	*b*∗
Control (wheat flour)	63.83 ± 0.24^A^	9.15 ± 0.12^A^	6.56 ± 0.39^A^
C (8%)-Ch (2%)+HPMC (0.3%)	58.73 ± 0.57^AB^	9.20 ± 0.13^A^	6.76 ± 0.36^A^
C (6%)-Ch (4%)+HPMC (0.3%)	57.56 ± 0.37^B^	9.26 ± 0.12^A^	7.03 ± 0.36^A^
C (4%)-Ch (6%)+HPMC (0.3%)	56.77 ± 0.61^B^	9.30 ± 0.18^A^	6.66 ± 0.18^A^
C (2%)-Ch (8%)+HPMC (0.3%)	55.02 ± 0.23^B^	9.20 ± 0.16^A^	6.50 ± 0.15^A^

C (8%)-Ch (2%): 8% corn flour and 2% chickpea flour; C (6%)-Ch (4%): 6% corn flour and 4% chickpea flour; C (4%)-Ch (6%): 4% corn flour and 6% chickpea flour; C (2%)-Ch (8%): 2% corn flour and 8% chickpea flour. ^∗^Data represent the mean ± standard deviation of three independent replications. ^∗∗^Different capital letters in each column indicate significant differences (*P* < 0.05).

**Table 5:** DSC of control sausage and gluten-free sausages modified by chickpea flour, corn flour, and HPMC.

Sample	To (°C)	Tp (°C)	Tf (°C)	Δ*H* (J/g dry sample)
Control (wheat flour)	106.25 ± 3.11^D^	119.23 ± 2.34^E^	177.76 ± 1.09^D^	108.33 ± 4.29^D^
C (8%)-Ch (2%)+HPMC (0.3%)	118.67 ± 2.04^C^	130.14 ± 0.32^D^	190.21 ± 0.59^C^	371.33 ± 3.41^C^
C (6%)-Ch (4%)+HPMC (0.3%)	117.23 ± 2.17^C^	132.19 ± 0.98^C^	189.11 ± 0.45^C^	368.95 ± 3.98^C^
C (4%)-Ch (6%)+HPMC (0.3%)	120.12 ± 0.76^B^	137.09 ± 1.12^B^	192.56 ± 0.34^B^	407.48 ± 12.13^B^
C (2%)-Ch (8%)+HPMC (0.3%)	122.34 ± 1.11^A^	140.71 ± 0.34^A^	194.31 ± 1.12^A^	519.11 ± 3.21^A^

C (8%)-Ch (2%): 8% corn flour and 2% chickpea flour; C (6%)-Ch (4%): 6% corn flour and 4% chickpea flour; C (4%)-Ch (6%): 4% corn flour and 6% chickpea flour; C (2%)-Ch (8%): 2% corn flour and 8% chickpea flour. ^∗^Data represent the mean ± standard deviation of three independent replications. ^∗∗^Different capital letters in each column indicate significant differences (*P* < 0.05).

**Table 6:** TGA of control sausage and gluten-free sausages modified by chickpea flour, corn flour, and HPMC.

Sample	∆*W*_1_ (mg)	∆*W*_2_ (mg)	∆*W*_3_ (mg)
Control (wheat flour)	89.12 ± 0.12^A^	84.38 ± 0.18^B^	70.29 ± 1.18^E^
C (8%)-Ch (2%)+HPMC (0.3%)	88.55 ± 0.46^AB^	87.48 ± 0.56^A^	83.82 ± 0.42^D^
C (6%)-Ch (4%)+HPMC (0.3%)	88.19 ± 0.23^BC^	87.30 ± 0.67^A^	85.95 ± 0.19^B^
C (4%)-Ch (6%)+HPMC (0.3%)	87.49 ± 0.19^C^	87.41 ± 0.49^A^	84.98 ± 0.32^C^
C (2%)-Ch (8%)+HPMC (0.3%)	86.10 ± 0.09^D^	88.17 ± 0.78^A^	87.55 ± 0.12^A^

C (8%)-Ch (2%): 8% corn flour and 2% chickpea flour; C (6%)-Ch (4%): 6% corn flour and 4% chickpea flour; C (4%)-Ch (6%): 4% corn flour and 6% chickpea flour; C (2%)-Ch (8%): 2% corn flour and 8% chickpea flour. ^∗^Data represent the mean ± standard deviation of three independent replications. ^∗∗^Different capital letters in each column indicate significant differences (*P* < 0.05).

**Table 7:** Textural properties of control sausage and gluten-free sausages modified by chickpea flour, corn flour, and HPMC.

Sample	Hardness (N)	Adhesiveness (mJ)	Chewiness (mJ)	Springiness (mm)	Gumminess (N)
Control (wheat flour)	2979 ± 78^E^	0.17 ± 0.06^C^	93.53 ± 1.12^D^	0.40 ± 0.02^A^	3139.3 ± 19.6^C^
C (8%)-Ch (2%)+HPMC (0.3%)	3240 ± 23^D^	0.27 ± 0.02^A^	128.89 ± 0.67^A^	0.42 ± 0.01^A^	3489.8 ± 22.4^A^
C (6%)-Ch (4%)+HPMC (0.3%)	3315 ± 11^C^	0.26 ± 0.01^A^	126.37 ± 0.34^B^	0.41 ± 0.00^A^	3470.0 ± 13.6^A^
C (4%)-Ch (6%)+HPMC (0.3%)	3774 ± 36^B^	0.25 ± 0.01^AB^	124.18 ± 0.78^C^	0.41 ± 0.01^A^	3445.5 ± 31.5^A^
C (2%)-Ch (8%)+HPMC (0.3%)	3936 ± 56^A^	0.22 ± 0.01^B^	123.55 ± 0.67^C^	0.40 ± 0.02^A^	3344.8 ± 23.7^B^

C (8%)-Ch (2%): 8% corn flour and 2% chickpea flour; C (6%)-Ch (4%): 6% corn flour and 4% chickpea flour; C (4%)-Ch (6%): 4% corn flour and 6% chickpea flour; C (2%)-Ch (8%): 2% corn flour and 8% chickpea flour. ^∗^Data represent the mean ± standard deviation of three independent replications. ^∗∗^Different capital letters in each column indicate significant differences (*P* < 0.05).

**Table 8:** Sensorial properties of control sausage and gluten-free sausages modified by chickpea flour, corn flour, and HPMC.

Sample	Color	Taste	Texture	Appearance	Overall
Control (wheat flour)	5.6 ± 0.7^A^	5.3 ± 0.6^A^	5.6 ± 0.5^A^	5.3 ± 0.2^A^	5.6 ± 0.5^A^
C (8%)-Ch (2%)+HPMC (0.3%)	5.2 ± 0.4^A^	4.8 ± 0.2^A^	5.0 ± 0.3^A^	5.4 ± 0.1^A^	5.4 ± 0.3^A^
C (6%)-Ch (4%)+HPMC (0.3%)	5.0 ± 0.3^A^	4.5 ± 0.1^A^	4.7 ± 0.2^A^	5.3 ± 0.2^A^	5.0 ± 0.2^A^
C (4%)-Ch (6%)+HPMC (0.3%)	4.6 ± 0.3^A^	4.6 ± 0.1^A^	4.5 ± 0.0^A^	5.5 ± 0.3^A^	4.8 ± 0.1^A^
C (2%)-Ch (8%)+HPMC (0.3%)	4.2 ± 0.1^B^	4.4 ± 0.0^B^	4.4 ± 0.0^B^	5.2 ± 0.2^A^	4.4 ± 0.1^B^

C (8%)-Ch (2%): 8% corn flour and 2% chickpea flour; C (6%)-Ch (4%): 6% corn flour and 4% chickpea flour; C (4%)-Ch (6%): 4% corn flour and 6% chickpea flour; C (2%)-Ch (8%): 2% corn flour and 8% chickpea flour. ^∗^Data represent the mean ± standard deviation of three independent replications. ^∗∗^Different capital letters in each column indicate significant differences (*P* < 0.05).

## Data Availability

The data used to support this study are available from the corresponding author upon request.
